# When Less Is Less: Solving Multiple Simple Problems Is Not Complex Problem Solving—A comment on Greiff et al. (2015)

**DOI:** 10.3390/jintelligence5010005

**Published:** 2017-01-05

**Authors:** Joachim Funke, Andreas Fischer, Daniel V. Holt

**Affiliations:** 1 Institute of Psychology, Heidelberg University, Hauptstr. 47, 69117 Heidelberg, Germany; daniel.holt@psychologie.uni-heidelberg.de; 2 Research Institute for Vocational Education and Training, Rollnerstraße 14, 90408 Nürnberg, Germany; fischer.andreas@f-bb.de

**Keywords:** complex problem solving, assessment, MicroDYN, MicroFIN, Genetics Lab, multiple complex systems, simulated microworlds, Tailorshop, validity

## Abstract

In this commentary, we critically review the study of Greiff, Stadler, Sonnleitner, Wolff, and Martin, “Sometimes less is more: Comparing the validity of complex problem solving measures” (*Intelligence,* **2015**, *50*, 100–113). The main conclusion of Greiff et al. that the “multiple complex systems” (MCS) approach to measuring complex problem-solving ability possesses superior validity compared to classical microworld scenarios (“less is more”) seems to be an overgeneralization based on inappropriate analysis and selective interpretation of results. In its original form, MCS is a useful tool for investigating specific aspects of problem solving within dynamic systems. However, its value as an instrument for the assessment of complex problem solving ability remains limited.

## 1. Introduction

As researchers involved in developing both the “minimal/multiple complex systems” (MCS) approach and the Tailorshop microworld simulation, we feel the need to comment on the article by Greiff et al. [1]. The main claim of Greiff et al. [1] is that administering a selection of tasks based on simple dynamic systems yields a measure of complex problem solving ability with superior validity compared to more complex classical microworld scenarios (“less is more”). We find that due to a number of problems in the statistical analysis and due to selective interpretation of results, conclusions about the true relation of MCS, the Tailorshop simulation, and the construct of complex problem solving presented in this article are not convincing. After discussing the limitations of the original article, we will present a conceptual analysis of the MCS approach and its relation to complex problemsolving ability. While we see potential in MCS as a tool for experimental research, we are skeptical about its suitability for assessment purposes, particularly in high-stakes situations such as educational testing or personnel selection. Considering the narrow conceptual base of MCS tests, such as MicroDYN or Genetics Lab, and the restricted range of criteria used to establish their external validity (mostly correlations with school grades), we conclude that the extent to which these tests can claim to measure a broad construct such as complex problem solving is limited. We will begin with a short summary of two of the main paradigms used in Greiff et al. [1] for readers unfamiliar with this line of research.

**Figure 1:**
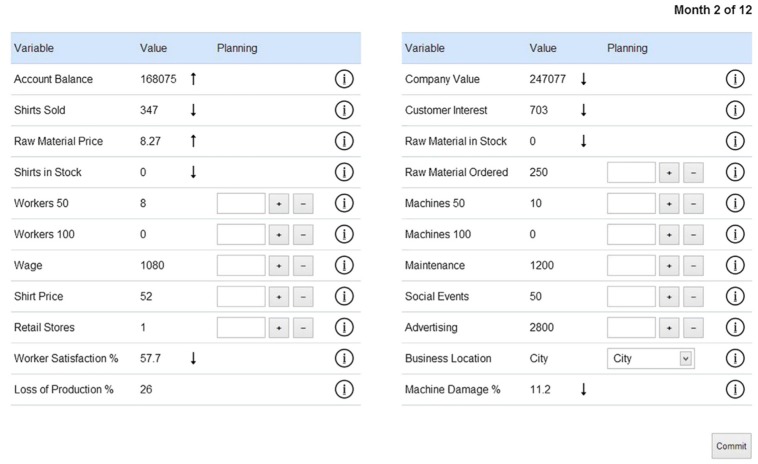
Screenshot of the Tailorshop’s graphical interface. Note the arrows indicating changes in the variables compared to the previous month.

In what has been called the “European tradition” of complex problem solving research [2], lay participants are faced with computer-simulated dynamic systems and given the task to explore and control these systems. In line with Greiff et al. [1], we will take “classical microworlds” to refer to computer-simulated scenarios of this type, such as the Tailorshop simulation and related tasks (e.g., LEARN, PowerPlant, FSYS; [3,4]), which are moderately complex and semantically rich. The Tailorshop (e.g., [5]) is a widely used microworld scenario in which participants take the role of the manager of a small textile company selling shirts (see Figure 1).

In the Tailorshop simulation participants can buy machines and materials, hire workers, set the sales price, decide how much to spend on advertising, and set several other variables in order to increase the value of the company. Participants are usually given six simulation cycles (“months” in the scenario) to freely explore the system, followed by a second run with 12 cycles in which to maximize company value [1,5,6]. To obtain more than a single measurement, the simulation can be run several times (e.g., [3,7]).

**Figure 2:**
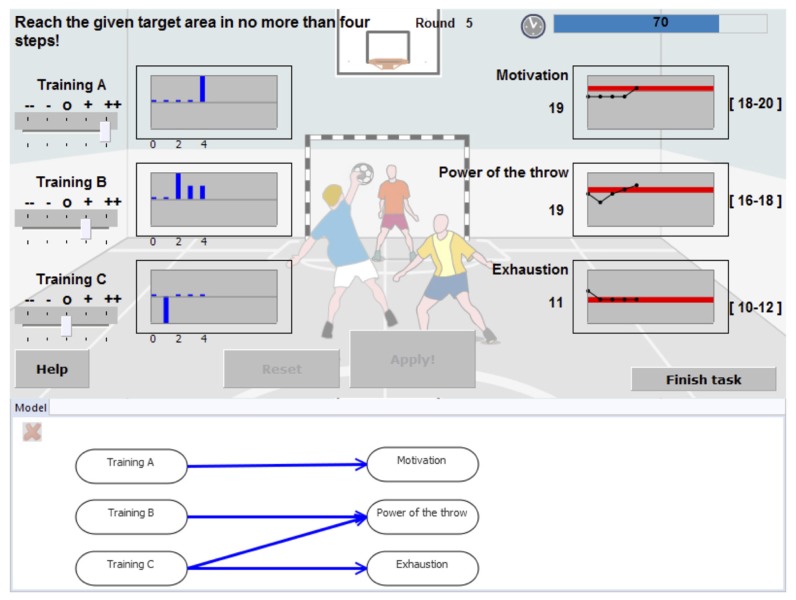
Screenshot of the MicroDYN item “Handball”: The participant has to find out how the input variables to the left are connected to the output variables on the right. Below the working area is an area for depicting the assumed causal structure.

In contrast, in MicroDYN [8] and Genetics Lab [9] (the most widely used MCS tasks), systems usually consist of just two or three input variables that can be manipulated by the participant and a similar number of output variables linked to the input variables by simple linear equations ([10]; see Figure 2). The semantic framing of MCS systems varies (e.g., chemistry lab, growing plants, or training handball players), but the semantics do not contain cues about the relations of system variables. In MicroDYN, the task is to first freely explore the system for a few minutes, draw a diagram of its structure and then control it for four simulation cycles. A test session usually involves exploration and control of several different systems. The label MCS for MicroDYN-type tests was first introduced as “*minimal* complex systems” in a German article by Greiff and Funke [11] (p. 218; also see [10]), referring to tasks based on simple linear structural equation systems (LSEs, Funke [12]). In recent articles, Greiff et al. [5,13] take MCS to mean “*multiple* complex systems”, which additionally includes problems based on finite state automata (e.g., MicroFIN).

## 2. Methodological Concerns

We agree with Greiff et al. [1] that a systematic comparison of different instruments for measuring complex problem solving ability and their relation to intelligence is missing. However, we do not see how this study can sufficiently fill this gap given that there are several methodological problems. These problems are: (1) reducing the broad class of classical microworlds to a single scenario; (2) ignoring the dependency structure of microworld performance indicators; and (3) applying highly circular reasoning for establishing construct validity.

The most obvious problem in Greiff et al. [1] is the reduction of the broad class of classical microworld scenarios to a *single task* (Tailorshop; [6]) with only *a single simulation run* used for assessing performance. This single measurement is in turn compared to *three* different MCS paradigms with a total of *28 tasks* (MicroDYN: 10 tasks, Genetics Lab: 16 tasks, MicroFIN: two tasks). While the Tailorshop is arguably a typical microworld task, it is questionable whether this setup allows a balanced comparison of the two approaches. The authors of [1] are aware of this asymmetry (p. 112) but do not consider it in most of their analysis and interpretation. Furthermore, including three separate MCS in the statistical analyses effectively gives MCS three chances to demonstrate superior validity compared to the single classical microworld. The use of only one single microworld task also contrasts with the plural term “classical measures (of CPS)” used repeatedly throughout the article, which suggests a breadth of approach that is simply not present in the design of the study. We agree that the Tailorshop is a good example of a classical microworld, but it seems unlikely that it can comprehensively cover the diverse group of simulated microworlds on its own (see [14] for an overview). While it is in principle possible to use a single measure to cover a broad construct, it is ultimately an empirical question whether any given test provides such comprehensive coverage. In the present study, no supporting evidence for this critical assumption is provided. On the contrary, extant research suggests that classical microworlds, including the Tailorshop, show little empirical overlap (e.g., [15,16,17]). This renders the choice of just a single scenario to represent performance in classical microworld simulations in a comparative analysis questionable. Previous research has shown how this problem can be avoided by using multiple different microworlds as indicators (e.g., [7,17]). At best the present article allows conclusions about a single microworld scenario, the Tailorshop, but due to other methodological problems, we think even this is uncertain.

Using only a single microworld run compared to three MCS tasks with 28 runs in total obviously introduces a reliability problem at the level of manifest test scores. Greiff et al. [1] try to counter this problem by using structural equation modeling, which can to some extent compensate differing reliabilities [18]. However, a proper application of this method requires multiple independent indicators per construct, which are not available for the classical microworld in the present study. Greiff et al. [1] therefore treat the cycles of the single microworld simulation run as items, which they group into “parcels” [19] to create three separate indicators. However, treating simulation cycles (or parcels of simulation cycles) within the same simulation run as independent indicators is ill-advised, as the cycles within a run are highly auto-correlated due to the nature of the scenario. As an extreme example, it is in principle possible to set up the simulated company in the Tailorshop optimally in the first simulation cycle and receive a perfect score for each subsequent cycle while doing nothing. This arguably warrants a maximum overall score for the complete run, but it is just as obvious that individual cycles cannot be treated as independent indicators of ability. In fact, how to handle this type of dependency problem is one of the main controversies in psychometric CPS research [16] and we co-developed the MCS approach as one way to address this problem by facilitating multiple short but independent simulation runs [10,11]. Greiff et al. [1] ignored the dependency problem in their analysis of the Tailorshop despite being aware of it [1] (p. 103: “… the changes between two months (i.e., the items) are hardly independent of each other.”).

In the present case, not considering the dependency structure is not only theoretically undesirable but appears to have a strong effect on the validity estimates for the latent variable constructed from the non-independent indicators. The system-induced auto-correlation of the simulation cycles inflates the reliability of the latent variable, as the (trait-unrelated) auto-correlation contributes to the true score estimate. This in turn attenuates the validity estimates of the resulting latent variable with respect to other constructs, which is exactly what can be observed in Greiff et al. [1]: The correlation of the latent classical microworld performance variable and a well-established marker of convergent validity, reasoning ability (Stadler et al. [20]), is just *r* = 0.24 (see [1], Table 2). For comparison, similar studies using structural equation modeling and the Tailorshop scenario find latent correlations of *r* = 0.76 [17] and *r* = 0.86 [7]. In terms of shared variance, this translates to a difference by about a factor of 10 between Greiff et al. [1] and the two reference studies (6% compared to 58% and 74%). This stark discrepancy with respect to a well-known marker of convergent validity begs an explanation and casts doubt on whether the latent variable formed in Greiff et al. [1] properly captures classical microworld performance. Ignoring the dependency structure of simulation cycles in the measurement model may well be an important part of the explanation 1. Unfortunately, it is not easily possible to model or separate out the effects of the dependency, as the exact auto-correlation structure of the system depends on the actions of participants. How to handle the dependency structure in dynamic systems used for psychometric assessment appropriately is an interesting question that deserves further research. It is therefore not clear whether this study can provide sufficient support for the superior predictive validity of multiple small complex systems (MCS) compared to classical microworlds (“less is more”).

Furthermore, the argument for the superior construct validity of MCS compared to classical microworlds (p. 105, [1]) seems to be that if three out of four tests (the MCS tests) correlate more highly with each other than with the fourth test (the Tailorshop), this is evidence for the construct validity of the MCS tests. Without further assumptions, this conclusion is not very strong, as the MCS tests establish their construct validity without reference to any external criteria. At most, we can conclude from the results that MCS tests and the Tailorshop seem to measure slightly different constructs, without any indication (beyond content validity) of what these constructs really are. Greiff et al. [1] refer to literature indicating that correlations among classical measures are generally lower than those among MCS tests. As we will argue in detail below, MCS tests may simply be conceptually narrow and therefore more similar (and hence more highly correlated) compared to the diversity of classical microworlds. What would be required for a genuine test of construct validity are appropriate, reliably measured, and relevant external criteria against which to validate new tests of CPS ability. Unfortunately, this is an area in which the psychometric approach to CPS in general is currently still lacking.

## 3. Selective Interpretation

Beyond the methodological problems described above, the interpretation of results in [1] seems somewhat selective. At first, the authors frankly acknowledge that “… contrary to our expectations, none of the MCS tests were significant predictors in the models [predicting science school grades] after we additionally controlled for reasoning.” (p. 111, [1]). In line with this statement, Table 2 (p. 109) shows that the most widely used MCS test (MicroDYN) has practically the same partial correlation with science school grades as the Tailorshop (*r* = 0.13 and *r* = 0.12, both non-significant) and even the best-performing MCS test in this study (MicroFIN) is not significantly different from the Tailorshop (*r* = 0.12 vs. *r* = 0.22, Fisher’s *z* = 0.80, *N* = 339, *p* = 0.18). However, the authors conclude (p. 111, emphasis added) that “[i]n summary, these results suggest that *MCS tests appear to be more valid than classical measures of CPS* in predicting real-world outcomes such as school grades.” That results are different when not controlling for reasoning seems a weak justification, as the authors rightly emphasize the importance of controlling for reasoning and have also done so in previous publications (e.g., [5,21]).

From our perspective, we conclude from the results reported in [1]: (a) the predictive validity of MCS tests is heterogeneous; (b) the Tailorshop microworld does about as well as the most widely used MCS test (MicroDYN); and (c) the surprise winner is a test based on finite state automata consisting of just two items (MicroFIN). The last finding casts doubt on a central tenet of the multiple complex systems approach, as two items seem a comparatively modest multiple by any standard. High reliability due to using multiple systems is good, but it is not a substitute for validity. Particularly given that the “classical measures” consisted of only a single microworld scenario, which was originally not developed for psychometric assessment, this is not evidence in favor of the MCS approach.

In addition, the presentation of the literature appears rather selective as well. In the introduction, evidence for the validity of classical microworlds is described as weak, yet no mention is made of the fact that except for predicting math and science school grades in some cases, there is little evidence for the validity of MCS. For example, taking job-related variables as a criterion, MicroDYN and MicroFIN did not show a significant relation to job complexity ratings, and only weak relations to “days of professional training per year” (*r* = 0.14) and income (standardized coefficient = 0.14) when controlling for reasoning [22,23]. For comparison, Danner et al. [6] and Kersting [15] report partial correlations of the Tailorshop and job performance ratings of *r* = 0.22 and *r* = 0.29 after controlling for reasoning. Similarly, the authors of [1] claim that “most of what is predictive of real-world outcomes in classical measures of CPS is accounted for by reasoning” (p. 104), but do not mention that in many cases controlling for reasoning or general cognitive ability reduces the predictive power of MCS tests to non-significance (e.g., MicroDYN in [1,24,25]). When intelligence is broadly operationalized this effect becomes even more pronounced [24]. Considering that recently a commercial test based on MicroDYN was published [26] and given that MicroDYN tasks have been used in large educational comparison studies such as PISA 2012 [27], a more balanced treatment of the evidence for the validity of these tests is desirable. From our perspective, a better summary with respect to external validity would be that both types of test can provide moderate increments over reasoning or intelligence, depending on the domain of validation and how reasoning or intelligence are operationalized. Perhaps unsurprisingly, MCS seem to have a slight edge for predicting science school grades, as many MCS are essentially scientific discovery tasks, while the Tailorshop has a slight edge when predicting professional success.

## 4. Conceptual Limitations

While we do think that there is value in the MCS approach as a research tool, we also think that it is important to acknowledge its limitations as an instrument for measuring complex problem solving as an ability. The main reason for this reservation is that we see a discrepancy between the broad, multi-faceted, and heterogeneous concept of complex problem solving on the one hand and the narrow conception of MCS tests on the other [28]. The simplicity of MCS is intended by design (hence the original label *minimal* complex systems) to facilitate focused research on specific aspects of CPS ([4,7]). However, when using MCS as a comprehensive psychometric approach to measuring CPS as an ability construct (e.g., [1,21,26]), this very simplicity becomes a problem. The defining attribute of complex problems is that they are complex—the exact opposite of simple. It is not evident how summing performance across multiple simple problems could result in a comprehensive measure of complex problem solving ability (as proposed in [1]). What MCS can provide are perhaps indices of specific subskills relevant for some CPS tasks (e.g., [29]). Labeling the resulting construct “complex problem solving” without any constraint seems an unwarranted overgeneralization. We would be open to revising this judgment if the proponents of using MCS as an assessment of CPS ability can provide evidence for the validity of their approach using reliably measured, adequately broad, and relevant external criteria.

The main conceptual problem for using MicroDYN-type MCS as a measure of CPS ability is that the structure of the items is very simple and that nearly all items can be solved using the same simple solution strategy. Thus, the construct measured by MicroDYN is likely to be narrow and may represent what is referred to in the literature as a *bloated specific*—a factor that arises simply from using highly similar items, resulting in high reliability at the expense of comprehensive construct coverage [30]. The restricted conceptual framework is both the strength and the weakness of MicroDYN. On the one hand, it facilitates the systematic construction of items with known properties; on the other hand, the items are very similar and can be solved using the same basic strategy. In essence, MicroDYN items are a variation of scientific discovery tasks ([26,31]) and can be solved by a “vary-one-thing-at-a-time” (VOTAT; [32,33]) strategy 2. As MicroDYN contains no stochastic elements, this simple strategy guarantees a perfect solution in practically all cases. Indeed, in [21] (p. 8) Wüstenberg et al. report a latent correlation of system knowledge acquired and using the vary-one-thing-at-a-time strategy in MicroDYN of *r* = 0.97, i.e., these facets of the test are practically identical. Furthermore, they found that it is only this factor (not control performance) that provides incremental validity over general intelligence when predicting school grades as an outcome measure (also see [34]). Wüstenberg et al. [35] furthermore found that a simple pen-and-paper knowledge test of VOTAT predicted the MicroDYN strategy to a significant extent. This supports that MicroDYN may simply measure a mix of intelligence and scientific reasoning skills, which fits with its incremental validity in respect of science school grades. While measuring scientific reasoning strategies such as VOTAT is interesting in its own right, particularly from an educational perspective (e.g., [32]), complex problem solving as a general construct cannot be reduced to VOTAT [28,34,36].

In addition to their simple structure, another feature of MCS tests is that they try to eliminate the effect of domain knowledge by using labels for system variables that do not provide cues about system relations (e.g., “handball training A, B, or C”). The goal is to separate the effect of domain knowledge from general problem solving ability. While the idea sounds convincing in principle, it is not clear whether this goal can be achieved and whether achieving it is desirable. First, even if the effect of domain-specific *content* knowledge can be controlled, the effect of domain-specific *strategy* knowledge remains, e.g., knowing how to conduct effective scientific exploration using VOTAT. Second, it is atypical for real-world complex problems to be knowledge-lean, instead handling information overload and intelligently using prior knowledge is typically part of solving many complex problems. Goode and Beckmann [37] have shown that prior system knowledge characteristically interacts with reasoning ability in controlling dynamic systems. It may be one of the characteristics of good problem solvers that they are able to effectively leverage their prior knowledge. Therefore, it is not clear whether trying to eliminate the effects of prior knowledge will lead to externally valid tests of CPS competence.

The narrow test concept of MCS also contrasts with the broad aspirations formulated by the authors. As a typical example for solving complex problems, Greiff et al. [1] (p. 101) describe a business consultant who restructures a company. How likely is it that a consultant in the real world will achieve this goal by simply “varying one thing at a time”? As has been argued elsewhere (e.g., [38,39]), highly specific analytical strategies, which may work in a restricted context (e.g., VOTAT in scientific laboratory–type situations), often fail in realistic situations even of only moderate complexity. This is not merely a problem of face validity but a challenge to the content validity and generalizability of MCS as a measure of complex problem solving ability. Primarily assessing a narrow facet of problem solving would also explain the unusually high internal consistency of MicroDYN-type tests compared to other problem solving tests and the mixed evidence for its validity [40]. The main consistent finding across different studies using MCS is a correlation with reasoning ability (see [20] for a review). However, the ambition of MCS is surely not to merely be an approximation of existing tests of reasoning ability. The main external criterion used so far is school grades, but why should school performance be a good indicator of complex problem solving ability? Compared to the complex business-, technology-, and politics-related examples that are often given as illustrations of CPS, school exercises are usually highly domain-specific with little in the way of general problem solving ability. Furthermore, assessing school grades by retrospective self-report with a delay of several years (as done in [1]) is also somewhat questionable when using them as the primary validation measure.

The examples given in the introduction of [1] suggest a particular closeness of MCS to job performance (e.g., secretary and business consultant), an important argument in other articles of these authors. However, in the literature we currently find only three published studies using MCS that involve samples from the working population. One of these only contains an uncontrolled mean comparison of blue collar workers with high school and university students, where the cause of group differences is uncertain [41]. In the second study, no relation of MCS performance to the level of job complexity coded according to the ICSO-8 standard (ILO [42]) was found; only a small relation to days of professional training per year was found (2% variance was when controlling cognitive ability, [22]). Finally, the results of Ederer et al. [23] also show only a small increment of MCS over other variables in predicting wages (1% variance explained, p. 442). The absence of effects or their relatively small magnitude (controlling for other factors) is surprising considering the implied importance of CPS for job performance. Furthermore, relevant criterion groups that should possess complex problem solving skills to a significant extent (e.g., business consultants, managers, political advisers, scientists) have not been investigated so far using MCS. At present, it seems an open question whether MCS tests provide a relevant increment over established measures for predicting real-world job performance. It appears Greiff et al. [1] may be right when they concede that ecological validity did not have priority in the design of MCS compared to other psychometric qualities (p. 105, [1]), although we think this is a debatable choice in an assessment context.

Finally, the narrow conception of MicroDYN-based tests makes the test prone to learning and training effects. Greiff et al. [5] (p. 592) acknowledge that training the test may be a problem for using MicroDYN, particularly in high-stakes testing situations such as personnel selection or educational testing. Given that a single simple strategy can be used for solving MicroDYN problems, it would be surprising if there were no learning effects between items and training-to-the-test should be comparatively easy. It is therefore not clear whether MicroDYN and its variants are suitable for (repeated) high-stakes testing in educational settings or personnel selection. While trainability is not explicitly addressed in the manual of the published MicroDYN-based test [26], it is potentially a serious issue that should be kept in mind when using tasks of this type for assessment purposes.

## 5. Conclusions and Outlook

To summarize our critique, we argue (a) that the analysis presented in Greiff et al. [1] has serious limitations; (b) that the interpretation of results and the presentation of existing literature is overly selective; and (c) that there are reasons to be skeptical about the value of MCS as a comprehensive approach to measuring CPS as an ability. As co-developers of MCS we feel that it has made a contribution to understanding the lower end of complexity in research on CPS. However, its potential as an assessment instrument remains limited. Tests based on minimal complex systems have a relatively narrow conceptual basis and evidence for external validity beyond school grades is scarce. As discussed above, the study by Greiff et al. [1] does not provide convincing evidence to assume otherwise. However, the development of a more broadly based battery of problem solving tests and improving the methodology for comparing measurement approaches may bring us closer to an understanding of the psychometric aspects of complex problem solving in the future.

The recent shift from “*minimal* complex systems” to “*multiple* complex systems” (in [1,5,13]) can be viewed as an attempt to broaden the MCS approach by including more heterogeneous problem types. While this is in principle a good idea, it gives up one of the greatest benefits of the original minimal complex systems approach, a unifying formal framework that guides the construction of items with predictable psychometric properties [10]. The only constraint of the new item type (MicroFIN) is that the systems underlying the items can be described using the formalism of finite state automata [5,43]. This formalism is so general that it can be used to implement literally any task, which renders the concept of multiple complex systems essentially arbitrary and provides little guidance for item construction (see p. 582, [5]). However, moving towards a carefully chosen battery of relatively heterogeneous complex tasks seems to be a good idea in principle to combine reliability and broad construct coverage, an approach that could perhaps be summarized as “more is more”.

From our perspective, complex problem solving is, first and foremost, a complex cognitive process, which involves a range of skills, abilities and knowledge. The goal of *process-oriented* CPS research is to understand the structure and mechanisms of this process (e.g., [14,44,45,46]). In contrast, *ability-oriented* CPS research (also called “psychometric CPS research”) focuses on individual differences in performance when carrying out this process. One approach to ability-oriented CPS research is based on the assumption of a uni- or multi-dimensional ability construct underlying CPS performance that is different from psychometric intelligence (e.g., [5,8,21]). An alternative ability-oriented approach considers CPS as a competency involving a heterogeneous mix of skills, abilities, knowledge and other factors which may vary by problem (e.g., [28,29,47]). In this view, CPS performance is what needs to be explained, for example by reference to existing individual-differences constructs. The approaches are complementary but place a different emphasis on how CPS as a construct is construed and investigated. Considering the evidence acquired over recent years, we think the notion of CPS as a generalizable ability construct different from psychometric intelligence is increasingly becoming questionable. Even some of the authors of [1] consider it an unanswered question whether CPS exists as a measurable attribute at all and whether current tests, including microworlds and MCS, are valid measures of CPS [48] 3.

We would like to conclude with several recommendations on how studies comparing the validity of complex problem solving assessments would ideally be conducted. There already are some good examples of how different aspects of this task can be approached (e.g., [6,7,17,24]). First, a balanced comparison of different CPS assessments requires an adequately broad sampling from the tests available for each measurement approach included (e.g., [17]); second, the problem of dependent items and learning effects needs to be handled appropriately, for example by modeling the dependencies, by selecting sufficiently different tests and test items, or by adapting administration and scoring procedures to reduce dependency ([7,17]); third, measurement reliability needs to be considered in the comparison, for example by using structural equation modeling [7,17,24]; fourth, for the assessment of external and construct validity, appropriate and reliably measured criteria are required that have an intrinsic relation to CPS. Particularly suitable criterion groups may be participants whose occupation involves CPS, such as managers, business consultants, political advisers, or scientists. While direct criterion group comparisons are a starting point, they are difficult to control for confounds (e.g., education). Another approach may be to employ measures of professional success (e.g., supervisor ratings, [6]). Fifth, to the extent that the increment of CPS competence over general cognitive ability is of interest, cognitive ability should be broadly operationalized using appropriate tests [24]. Additionally, for tests that involve specific domain knowledge, separate knowledge tests conducted before the simulation may be useful to control the interaction of domain knowledge with other aspects of CPS competence. Considering these suggestions to the extent practically possible may help to improve the quality of future studies investigating the validity of CPS assessments.

## Footnotes

1 Danner et al. [13] report the fit of a measurement model similar to Greiff et al. [1] but used a different model (which avoids the dependency problem) to determine latent correlations with other constructs (e.g., [5]).

2 For simplicity, we subsume the closely related “vary-nothing” strategy under the label VOTAT.

3 “Valid CPS assessments [...], whether in the form of CSMs [classical simulated microworlds] or MCSs, should be developed along the lines suggested by Borsboom et al. (2004) who state that “a test is valid for measuring an attribute if and only if (a) the attribute exists and (b) variations in the attribute causally produce variations in the outcomes of the measurement procedure” (p. 1061). *For CPS assessments, both questions remain unanswered for the time being.*” (Greiff and Martin [46], p. 2, emphasis added).
